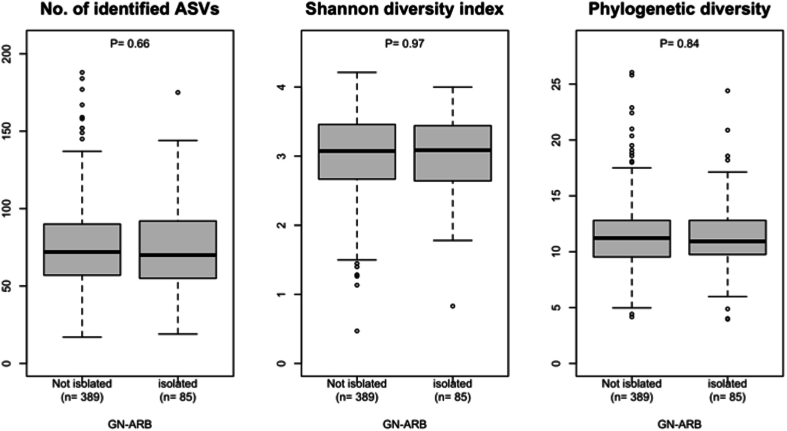# The oral cavity is a potential reservoir of gram-negative antimicrobial-resistant bacteria, which are correlated with ageing and the number of teeth

**DOI:** 10.1016/j.heliyon.2024.e39827

**Published:** 2024-10-28

**Authors:** Tomoki Kawayanagi, Miki Kawada-Matsuo, Toru Takeshita, Mi Nguyen-Tra Le, Mikari Asakawa, Yo Sugawara, Chika Arai, Kazuhisa Ouhara, Hiromi Nishi, Noriyoshi Mizuno, Hiroyuki Kawaguchi, Hideki Shiba, Motoyuki Sugai, Hitoshi Komatsuzawa

**Affiliations:** a Department of Biological Endodontics, Hiroshima University Graduate School of Biomedical and Health Sciences, Hiroshima, Japan; b Department of Bacteriology, Hiroshima University Graduate School of Biomedical and Health Sciences, Hiroshima, Japan; c Project Research Center for Nosocomial Infectious Diseases, Hiroshima University, Hiroshima, Japan; d Section of Preventive and Public Health Dentistry, Division of Oral Health, Growth and Development, Faculty of Dental Science, Kyushu University, Fukuoka, Japan; e Antimicrobial Resistance Research Center, National Institute of Infectious Diseases, Higashi Murayama, Japan; f Department of Periodontal Medicine, Hiroshima University Graduate School of Biomedical and Health Sciences, Hiroshima, Japan; g Department of General Dentistry, Hiroshima University Hospital, Hiroshima, Japan

## Abstract

**Objectives:**

The suppression of antimicrobial-resistant bacteria (ARB) is an important issue worldwide. In recent years, the presence of various ARB in the oral cavity has been reported, but the details remain unclear. Therefore, we aimed to isolate ARB from the oral cavity and investigate the factors affecting ARB colonization.

**Methods:**

Third-generation cephalosporin- or carbapenem-resistant gram-negative bacteria (GN-ARB) were isolated from the oral and nasal cavities of 514 participants who visited the dental clinic, and the whole-genome sequences of all the isolates were obtained. Additionally, the tongue microbiota was analysed by 16S rRNA sequencing. The correlations of GN-ARB isolation with clinical status and the tongue microbiota were subsequently investigated.

**Results:**

Among 514 participants, 131 and 13 GN-ARB strains were isolated from the oral cavities of 93 participants (18.1 %) and from the nasal cavities of 12 participants (2.3 %). The ARB were mainly affiliated with *Acinetobacter* spp. (39.7 %), *Pseudomonas* spp. (14.5 %) and *Stenotrophomonas maltophilia* (18.3 %). We found a correlation between the isolation of oral GN-ARB and ageing/the number of teeth. There were no significant correlations between the presence of GN-ARB and tongue microbiota composition.

**Conclusions:**

Our results suggest that the oral cavity is an important potential reservoir of GN-ARB and that ageing and tooth loss are risk factors for the presence of GN-ARB in the oral cavity.

## Highlights

• Colonization of antibiotic resistant Gram-negative bacteria in the oral cavity.
•Age and teeth number affect the oral colonization of antibiotic resistant bacteria.
•No relation of oral microflora with presence of antibiotic resistant bacteria.

## 1 Introduction

Antimicrobial-resistant bacteria (ARB) are a global concern, threatening the efficacy of chemotherapeutic agents against infectious diseases [1,2]. To combat ARB, the World Health Organization (WHO) has released a global action plan for antimicrobial resistance followed by national action plans in participating countries. As several bacterial species of ARB, such as methicillin-resistant *Staphylococcus aureus* (MRSA), vancomycin-resistant *Enterococcus* (VRE), extended spectrum β-lactamase (ESBL)-producing bacteria, and carbapenem-resistant *Enterobacterales* (CRE), are commonly found in humans, it is assumed that a certain percentage of people harbour ARB as commensal bacteria. Therefore, understanding the prevalence of commensal ARB in individuals with different backgrounds, such as patients in hospitals, residents in elderly care facilities, and persons living at home, is important. Recently, we reported that third-generation cephalosporin- or carbapenem-resistant gram-negative bacteria (GN-ARB), including ESBL-producing bacteria such as *Acinetobacter, Pseudomonas* and *Enterobacterales,* were isolated from the oral cavities of elderly persons in long-term care facilities (LTCFs) [3,4].

Interactions between oral bacteria and systemic diseases such as diabetes, arteriosclerosis, endocarditis, Alzheimer's disease, and cancer have been reported [[5], [6], [7]]. Therefore, oral bacteria are thought to spread throughout the whole body through blood vessels because they can enter blood vessels when bleeding occurs in cases of severe periodontitis, tooth extraction or periodontal surgery. In addition, aspiration pneumonia is a common disease in elderly individuals [8] and is one of the main causes of death, especially among older adults [8,9]. Many oral bacteria, such as oral streptococci, *Peptostreptococcus* and *Actinomyces*, have been identified in patients with aspiration pneumonia [10]. Additionally, *Enterobacterales, Pseudomonas aeruginosa, Staphylococcus aureus* and *Streptococcus pneumoniae*, as well as antibiotic-resistant bacteria, have been identified as causes of respiratory infectious diseases [11,12].

Based on these findings, examining the prevalence of ARB in the oral cavity in not only elderly in care facilities but also people living primarily in their own homes is important. Therefore, in this study, we attempted to isolate GN-ARB from the oral cavities of patients who visited the dental departments of Hiroshima University Hospital and investigated the correlations between demographic and oral status information and the presence of GN-ARB. In addition, we analysed the bacterial flora of the tongue and compared the composition of bacterial species between GN-ARB-positive and GN-ARB-negative patients.

## 2 Materials and methods

### 2.1 Study design

Samples were collected from August 2021 to December 2022 to assess the prevalence of GN-ARB with third-generation cephalosporin or carbapenem resistance in the oral and nasal cavities. A total of 514 patients (65 inpatients and 449 outpatients) visiting 7 dental departments at Hiroshima University Hospital were included in the study (Fig. S1). Informed consent was obtained from the patients, and specimens were collected. This study was approved by the ethics committee of the Hiroshima University Hospital Review Board (approval number E−2525) and the Kyushu University Committee of Ethics (approval number: M23109-00). All study procedures were performed in accordance with the Declaration of Helsinki. Informed consent was obtained from the study participants or their families prior to enrolment.

### 2.2 Sample collection

Specimens (oral and nasal samples) were collected from patients (including hospitalized patients) over 40 years of age who visited the dental outpatient clinic at Hiroshima University Hospital. Oral samples were collected from the patients prior to rinsing their mouths. Two samples were collected from the oral cavity. For the isolation of bacteria, sterile cotton swabs (Kawamoto Mekkin Swab #104, Hydraflox Swab 25-3706-H) were used to collect samples from the oral mucosa (i.e., the buccal mucosa, tongue, palate, and oral vestibule). For the analysis of bacterial composition, the membrane was set on an electric brush head (a modified Braun Oral-B Pro 500 electric toothbrush [Procter & Gamble, Cincinnati, OH]), and samples were taken from the surface of the tongue by rotating the membrane with the electric brush head as described previously [13,14]. Nasal samples were collected by wiping the mucosa of the nasal cavity. Cotton swabs were applied directly to two selective media, CHROMagar™ ESBL for ESBL-producing bacteria and CHROMagar™ mSuperCARBA™ medium plates for carbapenem-resistant bacteria (Kanto Chemical, Japan). The plates were incubated at 37 °C for 18–24 h, after which colony growth was observed. From each plate, a maximum of two colonies of different colour and morphology were selected. Selected colonies were cultured again on three plates: CHROMagar™ ESBL, CHROMagar™ mSuperCARBA™, and Candida GE plates. The isolates that grew on CHROMagar™ ESBL or CHROMagar™ mSuperCARBA™ but not on Candida GE were selected for further analysis. The samples used for microflora analysis (i.e., the membranes from tongue sampling) were immersed in lysis buffer (TE buffer with 0.1 % SDS) and stored at −80 °C until use.

### 2.3 Genomic analysis

The Illumina-generated whole-genome sequences of 144 clinical isolates were used for genome analysis. The extraction of genomic DNA, preparation of DNA libraries and paired-end sequencing were conducted as described elsewhere [3]. Isolates were cultured in Muller–Hinton broth overnight. Bacteria were lysed with 0.5 mg/mL lysozyme and 2 % SDS. Genomic DNA was purified from the lysate using AMPure XP beads (Beckman Coulter, USA). DNA libraries were prepared using an Enzymatics 5 × WGS fragmentation mix and WGS ligase reagents (Qiagen, Hilden, Germany), and paired-end sequencing (2 × 150 bp) was performed on the Illumina HiSeq X FIVE platform (Macrogen Japan Corporation, Tokyo, Japan) [15]. The bacterial species were determined with KmerFinder 3.2 (Center for Genomic Epidemiology: URL: https://cge.food.dtu.dk/services/KmerFinder/). We also analysed antibiotic resistance genes with ResFinder (Center for Genomic Epidemiology: URL: https://cge.food.dtu.dk/services/ResFinder/) [16]. The functions of the β-lactamases were analysed using a database (http://bldb.eu/Enzymes.php).

The genome data of the isolates and the sequence data of 16S rRNA evaluated in this study have been deposited in the DDBJ BioProject database as accession no. PRJDB17561 and PRJDB18419, respectively.

### 2.4 Clinical data

Among the 514 participants, clinical information was obtained from 480 participants, which included demographic information (age, sex, and inpatients/outpatients), oral conditions (the number of remaining teeth, obvious disease of the tongue and mucosa, and denture wearing) and prior use of antibiotics within one month. Periodontal examinations for PISA (periodontal inflamed surface area) and PESA (periodontal epithelial surface area) were evaluated in 170 participants. The PISA is a clinical index used to quantitatively evaluate the inflammatory area associated with periodontal disease and was developed by Nesse et al., in 2008 [17]. The PISA can be calculated based on the probing pocket depth (PPD) and bleeding on probing (BOP). All measurements were performed on fully erupted teeth at 6 sites per tooth using a Williams periodontal probe (HuFriedy, USA). Bleeding was recorded as either present or absent within 30 s of probing at 6 sites (mesiobuccal, mesiolingual, buccal, lingual, distobuccal, or distolingual) on each tooth. To calculate the PISA and PESA, data on PPD and BOP at six sites per tooth were entered into a spreadsheet that is accessible at http://www.parsprototo.info/docs/PISA_CAL.xls (free for use). The PESA includes an uninflamed periodontal epithelial surface; thus, the PISA/PESA ratio accurately reflects the degree of complete periodontal inflammation in each patient.

### 2.5 Tongue microbiota analysis

The bacterial composition of the tongue microbiota was determined by 16S rRNA gene sequencing analysis, as described previously [18]. DNA was extracted from tongue microbiota samples collected with a modified electric brush using the bead beating method. The V1 and V2 regions of the 16S rRNA gene in each sample were amplified using 8F (5′-AGA GTT TGA TYM TGG CTC AG-3′) and 338R (5′-TGC TGC CTC CCG TAG GAG T-3′) primers with sample-specific 8-base tags and adaptor sequences. PCR amplification, the purification of each amplicon, the pooling of amplicons, repurification, and quantification were performed as previously described [18]. Emulsion PCR was performed using an Ion One Touch 2 system (Thermo Fisher Scientific, Waltham, MA), and sequencing was performed on an Ion GeneStudio S5 system (Thermo Fisher Scientific).

Raw sequence reads were excluded from the analysis using R if they were ≤200 bases or ≥700 bases or if they did not include the correct forward and reverse primer sequences. The remaining reads were assigned to the appropriate sample by examining tag sequences using R, followed by the trimming of tag and primer sequences. These quality-checked reads were further processed using the DADA2 pipeline version 1.21.0 [19]. including quality-filtering, denoising, and chimera-filtering procedures, with default settings for Ion Torrent reads. Exact amplicon sequence variants (ASVs) present in each sample were defined in such procedures, and an ASV table was constructed. Ten ASVs observed in the negative control, which mainly corresponded to *Pseudomonas fluorescens*, were excluded from the ASV table and subsequently analysed as PCR contaminants. After rarefaction to 2500 reads, the number of amplicon sequence variants (ASVs), the Shannon diversity index, the phylogenetic diversity, and the weighted UniFrac metric [20] were calculated. Six samples with fewer than 2500 reads were excluded from the analyses. The taxonomy of each denoised sequence was determined using BLAST against 998 oral bacterial 16S rRNA gene sequences in eHOMD (eHOMD 16S rRNA RefSeq version 15.1) [21]. The nearest-neighbour taxon with ≥98.5 % identity was selected as a candidate for each sequence. The taxonomy of the remaining undefined sequences was determined at the genus level using the RDP classifier, with a minimum support threshold of 80 %.

Statistical analyses of the microbiota were conducted with R version 4.0.4. The Wilcoxon rank-sum test was used to compare the alpha diversity indices and the relative abundances of the predominant genera in the tongue microbiota. Differences in the bacterial composition of the tongue microbiota of patients with and without GN-ARB were assessed using PERMANOVA based on the weighted UniFrac metric. The detection rates of *Acinetobacter*, *Pseudomonas*, and *Stenotrophomonas* in the tongue microbiota according to 16S rRNA gene sequencing analysis were compared between patients with and without GN-ARB using Fisher's exact test.

### 2.6 Statistics

Correlations between clinical information and resistant bacteria were analysed by the chi-square test for categorical variables and the Mann‒Whitney *U* test for continuous variables. Multiple logistic regression analysis was performed for factors for which the analysis revealed p values less than 0.05 in the univariate analysis. Results with p values less than 0.05 were considered significant. All the statistical analyses, except for the tongue microbiota analysis, were conducted in JMP Pro version 17 (SAS Institute, Cary, NC, USA).

## 3 Results

### 3.1 Characteristics of the participants

In this study, samples were collected from 514 patients at Hiroshima University Hospital. Among these patients, 480 patients were included in the statistical analysis of their clinical information, and the mean age of the patients was 69.5 years (SD 11.3) (40–95 years). In terms of sex comparisons, there were 227 men (44.3 %), with a mean age of 69.8 ± 11.1 years (40–93 years), and 253 women (52.7 %), with a mean age of 69.2 ± 11.4 years (40–95 years).

### 3.2 Isolation of GN-ARB from the oral and nasal cavities

Among the 514 participants, 100 had GN-ARB in the oral or nasal cavity (19.5 %) (Fig. 1a). Eighty-eight (17.1 %) participants had GN-ARB in only the oral cavity, 7 (1.4 %) participants had GN-ARB in only the nasal cavity, and 5 (1.0 %) partcipants had GN-ARB in both cavity sites. Five participants had ESBL-producing bacteria in the oral cavity, whereas 48 and 5 participants had carbapenemase (CPase)-producing bacteria in the oral and nasal cavities, respectively. The isolation ratio of GN-ARB from the oral and nasal cavities among the 7 dental departments was not significantly different (Table S1).

**Fig. 1:**
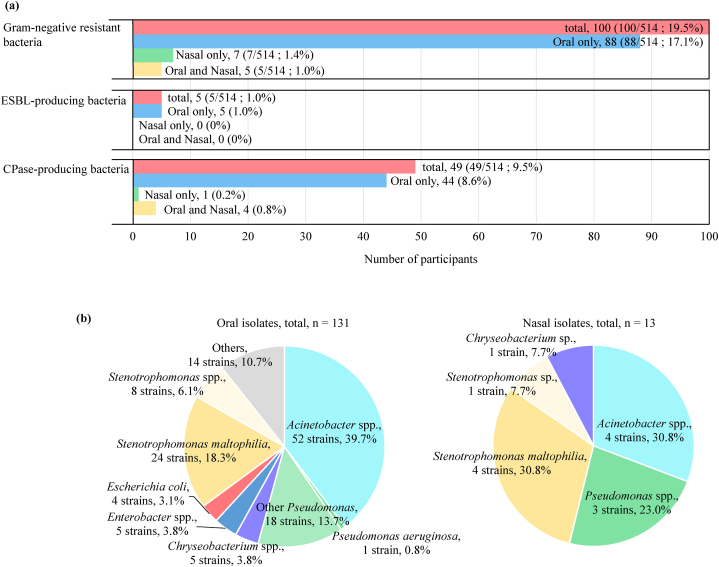
Isolation of gram-negative ARB from oral and nasal cavities. (a) Number of participants with gram-negative ARB isolated from the oral and nasal cavities. (b) Percentages of genera among gram-negative ARB in the oral and nasal cavities.

The distribution of the bacterial genera associated with GN-ARB is shown in Fig. 1b. In total, 131 and 13 isolates were obtained from the oral and nasal cavities, respectively. In the oral cavity, *Acinetobacter* (52 isolates, 39.7 %; 9 species), *Stenotrophomonas* (32 isolates, 24.4 %; 3 species) and *Pseudomonas* (19 isolates, 14.5 %; 6 species) were commonly isolated. In the nasal cavity, only 13 isolates were found, including 4 *Acinetobacter* isolates, 5 *Stenotrophomonas* isolates, 3 *Pseudomonas* isolates and 1 *Chryseobacterium* isolate. All bacterial species isolated from the oral and nasal cavities are listed in Table S2.

Five ESBL-producing isolates, including *E. coli* and *Elizabethkingia* sp., were found in the oral cavity (Table 1). The ESBL genes were *bla*_CTX-M-15_ in 1 *E. coli* isolate, *bla*_CTX-M-27_ in 3 *E. coli* isolates, *bla*_TEM-1B_ in 1 *E. coli* isolate and *bla*_CME-1_ in 1 *Elizabethkingia* sp. isolate. Among the 57 CPase-producing isolates, 30 *Stenotrophomonas* spp. isolates, 15 *Acinetobacter* spp. isolates, 5 *Chryseobacterium* spp. isolates, 1 *Elizabethkingia* sp. isolate and 1 *Pseudomonas* sp. isolate were found in the oral cavity, whereas 4 isolates of *Stenotrophomonas* sp. and one isolate of *Chryseobacterium* sp. were found in the nasal cavity (Table 1). The CPase genes were identified as *bla*_OXA_ types in *Acinetobacter* isolates, *bla*_IND_ types in *Chryseobacterium* sp., *bla*_IND_ and *bla*_GOB_ types in *Elizabethkingia, bla*_IND_ types in *Pseudomonas* sp.*, and bla*_L1_ in *Stenotrophomonas* isolates.

**Table 1:** ESBL and CPE genes in Cephalosporin/Carbapenem-resistant isolates by bacterial species from oral and nasal cavity.

species	strain	ESBL	CPE	species	strain	ESBL	CPE
*A. baumannii*	HN74 (O)^a^	–	*bla*_OXA-510_	*C. arthrosphaerae*	HN258 (O)	–	*bla*_IND-3_
	HN228 (O)	–	*bla*_OXA-65_	*C. indologenes*	HN283 (O)	–	*bla*_IND-2b_
	HN320 (O)	–	*bla*_OXA-98_		HN289 (O)	–	*bla*_IND-2b_
	HN341 (O)	–	*bla*_OXA-98_	*Chryseobacterium* sp.	HN158 (N)	–	*bla*_IND-7_
*A. bereziniae*	HN44 (O)	–	*bla*OXA-355		HN274 (O)	–	*bla*_IND-8 like_
	HN69 (O)	–	*bla*_OXA-355_		HN300 (O)	–	*bla*_IND-4_
*A. oleivorans*	HN214 (O)	–	*bla*_OXA-305_	*Elizabethkingia* sp.	HN13 (O)	*bla*_CME-1_	*bla*_GOB-13_ *bla*_B-9_
	HN310 (O)	–	*bla*_OXA-304_	*E. coli*	HN48 (O)	*bla*_CTX-M27_	–
*A. pittii*	HN33 (O)	–	*bla*_OXA-417_		HN88 (O)	*bla*_CTX-M27_ *bla*_TEM-1B_	–
	HN60 (O)	–	*bla*_OXA-272_		HN210 (O)	*bla*_CTX-M15_	–
	HN201 (O)	–	*bla*_OXA-272_		HN219 (O)	*bla*_CTX-M27_	–
	HN281 (O)	–	*bla*_OXA-272_	*Pseudomonas* sp.	HN154 (O)	–	
*A. radioresistens*	HN1 (O)	–	*bla*_OXA-565_	*S. maltophilia*	28 isolates	–	
*Acinetobacter* sp.	HN308 (O)	–	*bla*_OXA-270_		(O:24, N:4)	–	
	HN322 (O)	–	*bla*_OXA-355_	*S. pavanii*	4 isolates (O)	–	
				*Stenotrophomonas* sp.	2 isolates (O)	–	

a O and N represent Oral and Nasal, respectively.

### 3.3 Associations between clinical characteristics and GN-ARB isolation

For statistical analysis, we analysed the correlation between GN-ARB in the oral cavity/nasal cavity and the clinical status of patients (Table 2). There were significant correlations between age/the number of current teeth/dentures used and the presence of oral GN-ARB. However, we did not find any correlations with nasal GN-ARB. Logistic regression analysis further demonstrated that the number of teeth (10–23 teeth) and age were significantly associated with the presence of GN-ARB in the oral cavity after controlling for the effects of confounding factors (Table 3).

**Table 2:** Descriptive statistics of analytes with gram-negative ARB in the oral and nasal cavities.

	Subjects with gram-negative ARB in oral cavity			Subjects with gram-negative ARB in nasal cavity		
	Negative	Positive	*p*-value^a^	Negative	Positive	*p*-value^a^
	(n = 393)	(n = 87)		(n = 468)	(n = 12)	
Age	68.7 ± 11.5	73.1 ± 9.1	<0.001	69.6 ± 11.3	66.5 ± 9.8	0.25
Sex			0.81			0.56
Male	187 (47.6 %)	40 (46.0 %)		220 (47.0 %)	7 (58.3 %)	
Female	206 (52.4 %)	47 (54.0 %)		248 (53.0 %)	5 (41.7 %)	
Impatient or outpatient			0.73			0.66
Outpatient	340 (86.5 %)	77 (88.5 %)		407 (87.0 %)	10 (83.3 %)	
Impatient	53 (13.5 %)	10 (11.5 %)		61 (13.0 %)	2 (16.7 %)	
Use of antimicrobials within 1 month			0.74			0.70
Yes	62 (15.8 %)	12 (13.8 %)		73 (15.6 %)	1 (8.3 %)	
No	331 (84.2 %)	75 (86.2 %)		395 (84.4 %)	11 (91.7 %)	
With remaining teeth (Continuous)	22.0 ± 7.4	20.0 ± 7.2	<0.001	21.8 ± 7.3	17.6 ± 11.2	0.31
With remaining teeth (Category)			0.0024			0.023
≧24	224 (57.0 %)	32 (36.8 %)		250 (53.4 %)	6 (50.0 %)	
10〜23	135 (34.4 %)	44 (50.6 %)		177 (37.8 %)	2 (16.7 %)	
≦9	34 (8.6 %)	11 (12.6 %)		41 (8.8 %)	4 (33.3 %)	
Obvious diseases of the tongue and oral mucosa			0.082			1.00
Yes	37 (9.4 %)	14 (16.1 %)		50 (10.7 %)	1 (8.3 %)	
No	356 (90.6 %)	73 (83.9 %)		418 (89.3 %)	11 (91.7 %)	
Denture use or not			0.032			1.00
Yes	119 (30.3 %)	37 (42.5 %)		152 (32.5 %)	4 (33.3 %)	
No	274 (69.7 %)	50 (57.5 %)		316 (67.5 %)	8 (66.7 %)	

a Fisher's exact test was performed for categorical variables, and Mann-Whitney *U* test was performed for continuous variables.

**Table 3:** Correlation of oral Gram-negative ARB carriage with patient background factors.

	Univariate Logistic Regression model		Multivariate Logistic Regression model^a^	
	Crude odds ratio (95 % CI)	*p*-value	Adjusted odds ratio (95 % CI)	*p*-value
Age	1.04 (1.02–1.06)	<0.001	1.03 (1.00–1.05)	0.036
Sex	0.94 (0.59–1.49)	0.79	0.87 (0.54–1.41)	0.57
With remaining teeth				
24≧	1.00		1.00	
10〜23	2.28 (1.38–3.77)	0.0013	1.83 (1.06–3.16)	0.029
≦9	2.26 (1.04–4.91)	0.039	1.73 (0.75–4.00)	0.20

95 % CI, 95 % confidence intervals.

a each odds ratio was adjusted by age, sex and with remaining teeth.

We hypothesized that tooth loss would be associated with the detection of GN-ARB in the oral cavity via gingival inflammation due to periodontitis. We compared the periodontal conditions of 170 patients who received detailed periodontal conditions compatible with the PISA and PESA, and the patients included those with and without GN-ARB in the oral cavity (Fig. S2). However, no significant difference was observed in the PISA/PESA ratio between patients with and without GN-ARB in the oral cavity (P = 0.33, Mann‒Whitney *U* test).

### 3.4 Comparison of the composition of tongue microbiota

We subsequently hypothesized that the emergence of GN-ARB in the oral cavity would be induced by disruption of the normal indigenous microbiota in the oral cavity. To test this hypothesis, we determined the bacterial composition of the tongue microbiota of each patient using 16S rRNA sequencing analysis and compared the results from patients with GN-ARB to those from patients without GN-ARB. However, beta diversity analysis based on the weighted UniFrac metric revealed that the overall bacterial composition of the tongue microbiota of patients with GN-ARB was not significantly different from that of patients without GN-ARB (P = 0.29, PERMANOVA; Fig. 2a). No significant differences were observed in any of the three alpha diversity indices between the samples from patients with GN-ARB and those from patients without GN-ARB (Fig. S3). The tongue microbiota compositions of the patients in both groups were dominated by 12 bacterial genera, such as *Neisseria, Streptococcus,* and *Prevotella* (mean relative abundances >1 %), and no significant difference was observed in their relative abundances between the patients with GN-ARB and those without GN-ARB (Fig. 2b). *Acinetobacter*, *Pseudomonas*, and *Stenotrophomonas* GN-ARB genera isolated in this study were more frequently detected in patients with GN-ARB than in those without GN-ARB (Table S3). However, these GN-ARB were identified in several patients only by 16S rRNA gene sequencing analysis, suggesting that these GN-ARB comprise a minority among the tongue microbiota and that their relative abundances are often lower than the detection threshold of 16S rRNA gene analysis.

**Fig. 2:**
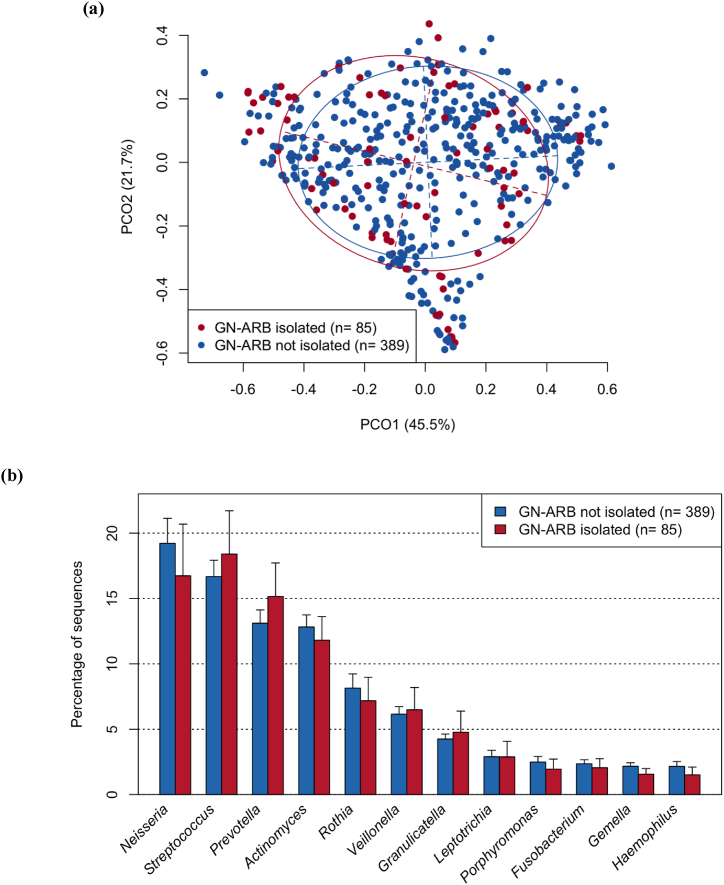
Comparison of bacterial flora in patients with or without GN-ARB in the oral cavity. (a) Principal coordinate analysis (PCoA) plot showing similarity relationships among tongue microbiota constituents from the 484 patients according to the weighted UniFrac distance metric. The samples of patients with and without GN-ARB are depicted as dots in different colours (GN-ARB isolated and GN-ARB not isolated, respectively). The ellipses cover 67 % of the samples belonging to each group. The axes explain 45.5 and 21.7 % of the variance. Significant differences were assessed using the Wilcoxon rank-sum test with Benjamini–Hochberg adjustment for multiple comparisons. (b) Relative abundances of predominant bacterial genera (mean relative abundance >1 %) in patients with and without GN-ARB in tongue microbiota samples (GN-ARB isolated and GN-ARB not isolated, respectively).

No significant difference was observed in the total relative abundances of the 10 periodontitis-associated taxa identified by Oliveira et al. [22] between patients with GN-ARB (0.26 ± 1.04 %, mean ± SD) and those without GN-ARB (0.17 ± 0.96 %; P = 0.32, Wilcoxon rank-sum test); these data support the hypothesis that periodontitis is unlikely to be associated with the detection of GN-ARB in the oral cavity.

## 4 Discussion

In this study, we isolated third-generation cephalosporin/carbapenem-resistant gram-negative bacteria from dental patients ranging from 40– to 90 years of age. Previously, in 2 different investigations [4,23], we isolated third-generation cephalosporin/carbapenem-resistant GN-ARB from residents of long-term care facilities. Among 98 residents, 37.8 % had these resistant bacteria [23], and among 178 residents, 52 residents (29.2 %) had resistant bacteria [4]. Therefore, the rate of isolation from dental patients in this study (19.5 %) was lower than that from residents of long-term care facilities. Because we found a correlation between age and GN-ARB in this study, we believe that this difference could be because the average ages of the participants in the studies by Le et al. [23] and Haruta et al. [4] and in this study were 83.3 ± 9.5 years, 87.0 ± 9.1 years and 69.7 ± 12.8 years, respectively. However, when the target population was narrowed down to those older than 80 years in this study, 21 of the 88 participants (83.7 ± 3.5 years) had GN-ARB, resulting in an isolation rate of 23.9 %, which was still lower than the isolation rate in elderly care facilities. Based on these investigations, we propose that the prevalence of ARB in residents of LTCFs is high due to the following risk factors: resident characteristics such as old age, comorbidities and underlying diseases, and environmental factors such as the use of gastrointestinal devices, mechanical ventilation, and the ability to access a room with carriers in the same ward. As the participants in this study were mostly outpatients (417/480 participants), they had fewer risk factors than did the residents of LTCFs. However, importantly, a certain percentage (19.5 %) of dental patients with fewer risk factors also harboured GN-ARB. Considering nosocomial infection control, health care workers need to understand that the oral cavity is a reservoir for ARB and that there is a possibility of ARB in the oral cavities of members of the general population.

Among 514 patients, 144 GN-ARB, including 5 ESBL-producing bacteria and 57 CPase-producing bacteria, were isolated (Table 1). The oral isolation rate of the ESBL-producing strains in this study (0.97 %) was significantly lower than those reported in a previous study (*Enterobacterales*: 5.6 %, *P. aeruginosa:* 3.4 %) assessing LTCFs [3,4]. Among the 5 ESBL-producing bacteria, 4 *E. coli* isolates, 3 with *bla*_CTX-M-27_ and 1 with *bla*_CTX-M-15_, were categorized as ST131 *fimH*30*,* which has been reported to be the predominant high-risk clone worldwide [24,25]. Therefore, our findings indicate that a global clone of *E. coli* ST131 could be present in the oral cavities of not only inpatients and residents of LTCFs but also the general public (dental outpatients). Among 144 GN-ARB strains, 57, including 34 *Stenotrophomonas* isolates, possessed CPase genes. *S. maltophilia* produces two β-lactamases, L1 class B metallo-β-lactamases, which hydrolyse most β-lactams, including penicillins, cephalosporins and carbapenems, and L2 class A serine cephalosporases, which hydrolyse penicillins, cephalosporins and monobactams [26]. Therefore, *S. maltophilia* shows natural resistance to carbapenem. In addition to *Stenotrophomonas*, some *Acinetobacter* spp. (15 of 56 isolates; 26.8 %) had *bla*_OXA_-type CPases, whereas others did not. Carbapenem resistance is mediated by multiple factors, including CPase activity, efflux pumps and porin mutations [27,28]; thus, the absence of CPase genes in the isolates in this study may be related to the presence of other resistance factors.

Importantly, we found correlations between the prevalence of GN-ARB and age and between the prevalence of GN-ARB and the number of teeth (Table 2, Table 3). The greater the age and the lower the number of teeth, the higher the rate of oral colonization by GN-ARB. Compared with younger people, older people have many diseases, such as aspiration pneumonia, urinary tract infection, intra-abdominal sepsis, bacterial meningitis, infective endocarditis, septic arthritis, and tuberculosis [29]. As a consequence, antibacterial agents are frequently used by older people. This usage promotes the emergence and spread of ARB among older people. Therefore, the correlation between the prevalence of GN-ARB and age is reasonable, although this is the first report focusing on GN-ARB in the oral cavity. Given that the relationship between age and the number of teeth is generally considered, we subsequently performed multiple logistic regression analysis and found that age was strongly correlated with the number of remaining teeth from 10 to 23 teeth and that age was related to the remaining teeth in individuals with <9 teeth (Table 3). Therefore, we propose that the isolation of GN-ARB is correlated with the number of teeth, independent of age. This is the first report of the importance of the number of teeth with respect to the presence of ARB in the oral cavity. Generally, tooth loss is caused by periodontal disease. Therefore, we evaluated the oral condition of patients by the PISA/PESA and compared the PISA/PESA ratios between GN-ARB-positive and GN-ARB-negative patients; however, we found no correlation between them (Fig. S2). In addition, the analysis of bacterial flora also revealed no significant trends in identifying periodontopathogenic bacteria such as *Porphyromonas* and *Prevotella*. Based on these results, we concluded that GN-ARB isolation was not directly associated with current periodontal conditions.

Previously, several analyses of the role of the oral microbiota in ageing have been reported [30,31]. However, there are no consensus findings concerning the characteristics of the oral microbiota at various ages. Kazarina A reported that, compared with individuals 40–60 years old and older, those 20–40 years old had the highest bacterial diversity in supragingival plaque samples [31]. Additionally, the abundance of the oral commensal *Neisseria* decreased after age 40, and the prevalence of *Streptococcus anginosus* and *Gemella sanguinis* gradually increased with age. Liu et al. reported that bacterial α diversity decreased with age, whereas β diversity slightly increased in 3 samples (gingival crevicular fluid, tongue back and saliva) [30]. In this study, we detected no notable difference in overall microbiota composition between tongue microbiota samples with and without GN-ARB. Based on these results, we speculate that a decreased number of teeth may cause dynamic or niche changes in the oral environment, thereby allowing GN-ARB infection. In addition, three genera, *Acinetobacter, Pseudomonas, Stenotrophomonas*, mainly isolated in this study were detected from only a small number of the participants via the 16S rRNA gene sequencing analysis (Table S3), indicating that they are minority members in the oral microbiota, which is constituted by numerous commensals.

There are some limitations in the present study. This study examined ARB isolation in dental outpatients. Since most dental outpatients live at home, we suppose that the results of this study reflect the ARB isolation rates in the general population. However, the population of dental patients may not accurately reflect the general population. In addition, it was impossible to set a defined timing, such as at the time of the initial visit, at the time of treatment, or at the time of follow-up, for taking the samples from these patients.

In conclusion, our results indicate that more attention should be given to oral ARB for the prevention of ARB-related diseases, and these results demonstrate the importance of maintaining oral health as a preventive measure such as professional mechanical tooth cleaning and the use of mouth wash containing disinfectant.

## CRediT authorship contribution statement

**Tomoki Kawayanagi:** Writing – review & editing, Writing – original draft, Visualization, Investigation, Formal analysis, Data curation. **Miki Kawada-Matsuo:** Writing – review & editing, Writing – original draft, Validation, Resources, Project administration, Methodology, Investigation, Funding acquisition, Formal analysis, Conceptualization. **Toru Takeshita:** Writing – review & editing, Writing – original draft, Visualization, Investigation, Formal analysis, Conceptualization. **Mi Nguyen-Tra Le:** Writing – review & editing, Visualization, Validation, Software, Resources, Methodology, Formal analysis, Data curation. **Mikari Asakawa:** Writing – review & editing, Investigation, Formal analysis. **Yo Sugawara:** Writing – review & editing, Investigation. **Chika Arai:** Writing – review & editing, Investigation. **Kazuhisa Ouhara:** Writing – review & editing, Investigation. **Hiromi Nishi:** Writing – review & editing, Investigation. **Noriyoshi Mizuno:** Writing – review & editing, Supervision, Investigation. **Hiroyuki Kawaguchi:** Writing – review & editing, Supervision, Investigation. **Hideki Shiba:** Writing – review & editing, Supervision, Resources, Conceptualization. **Motoyuki Sugai:** Writing – review & editing, Resources, Methodology, Conceptualization. **Hitoshi Komatsuzawa:** Writing – review & editing, Writing – original draft, Validation, Supervision, Resources, Project administration, Methodology, Investigation, Funding acquisition, Conceptualization.

## Funding information

This study was supported by The Japan Agency for Medical Research and Development (AMED) under Grant Number JP23fk0108606.

## Declaration of competing interest

The authors declare the following financial interests/personal relationships which may be considered as potential competing interests:Hitoshi Komatsuzawa reports financial support was provided by 10.13039/100009619The Japan Agency for Medical Research and Development (10.13039/100009619AMED). If there are other authors, they declare that they have no known competing financial interests or personal relationships that could have appeared to influence the work reported in this paper.

## Figures and Tables

**figs1:**
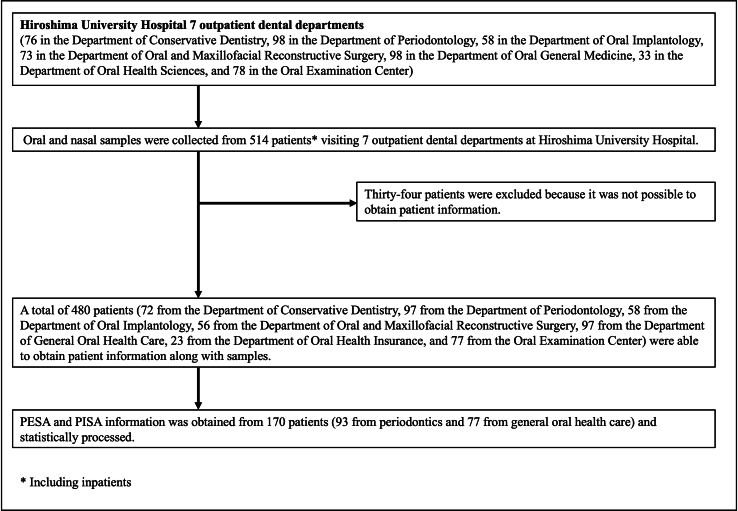


**figs2:**
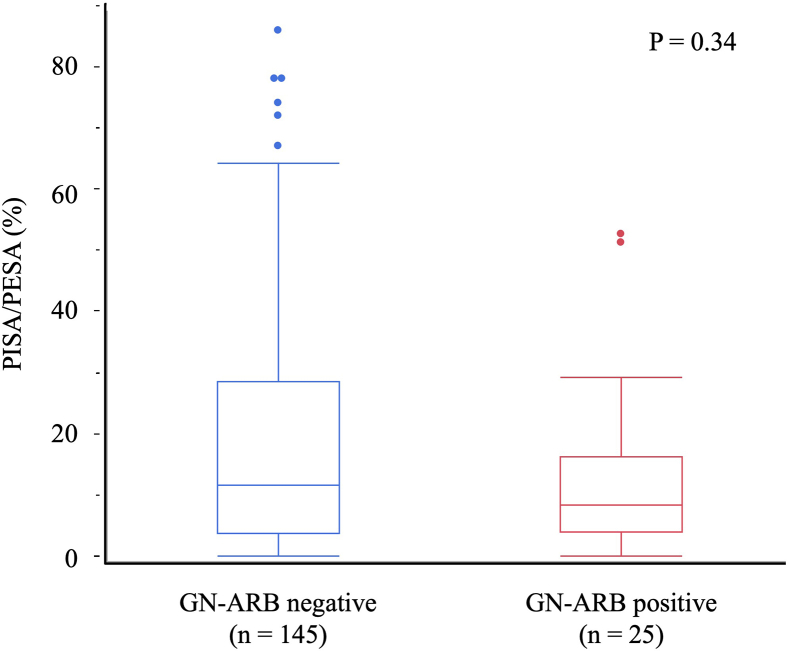


**figs3:**