# Supplementary material for: The oral cavity is a potential reservoir of gram-negative antimicrobial-resistant bacteria, which are correlated with ageing and the number of teeth
Source: Heliyon. 2024 Oct 28;10(21):e39827. doi: 10.1016/j.heliyon.2024.e39827 (PMC11565020; doi:10.1016/j.heliyon.2024.e39827)
Supplement: Multimedia component 1 [file mmc1.docx]

**Table S1. Comparison of the isolation ration of Gram-negative ARB among 7 dental departments**

|  | **Subjects with gram-negative ARB  in oral cavity** | | | **Subjects with gram-negative ARB  in nasal cavity** | | |
| --- | --- | --- | --- | --- | --- | --- |
|  | **Negative** | **Positive** | ***p*-value^a^** | **Negative** | **Positive** | ***p*-value^a^** |
|  | **(n = 393)** | **(n = 87)** |  | **(n = 468)** | **(n = 12)** |  |
|  |  |  | 0.094 |  |  | 0.24 |
| A | 67 (17.1%) | 5 (5.8%) |  | 69 (14.7%) | 3 (25.0%) |  |
| B | 79 (20.1%) | 18 (20.7%) |  | 92 (19.7%) | 5 (41.6%) |  |
| C | 45 (11.5%) | 13 (15.0%) |  | 58 (12.4%) | 0 (0.0%) |  |
| D | 41 (10.4%) | 15 (17.2%) |  | 54 (11.5%) | 2 (16.7%) |  |
| E | 80 (20.4%) | 17 (19.5%) |  | 95 (20.3%) | 2 (16.7%) |  |
| F | 19 (4.8%) | 4 (4.6%) |  | 23 (4.9%) | 0 (0.0%) |  |
| G | 62 (15.7%) | 15 (17.2%) |  | 77 (16.5%) | 0 (0.0%) |  |

^a^Fisher's exact test.
