# Supplementary material for: The oral cavity is a potential reservoir of gram-negative antimicrobial-resistant bacteria, which are correlated with ageing and the number of teeth
Source: Heliyon. 2024 Oct 28;10(21):e39827. doi: 10.1016/j.heliyon.2024.e39827 (PMC11565020; doi:10.1016/j.heliyon.2024.e39827)
Supplement: Multimedia component 2 [file mmc2.docx]

**Table S2. Number of and Cephalosporin/Carbapenem-resistant isolates by bacterial species**

**from oral and nasal cavity**

|  |  | Oral |  | Nasal |
| --- | --- | --- | --- | --- |
| *Achromobacter insolitus* |  | 1 |  |  |
| *Achromobacter* sp. |  | 1 |  |  |
| *Acinetobacter baumannii* |  | 4 |  |  |
| *Acinetobacter bereziniae* |  | 2 |  |  |
| *Acinetobacter nosocomialis* |  | 6 |  | 1 |
| *Acinetobacter oleivorans* |  | 2 |  |  |
| *Acinetobacter pittii* |  | 4 |  |  |
| *Acinetobacter radioresistens* |  | 1 |  |  |
| *Acinetobacter seifertii* |  | 5 |  | 1 |
| *Acinetobacter soli* |  |  |  | 1 |
| *Acinetobacter ursingii* |  | 26 |  | 1 |
| *Acinetobacter* sp. |  | 2 |  |  |
| *Brucella intermedia* |  | 1 |  |  |
| *Burkholderia* sp. |  | 1 |  |  |
| *Chryseobacterium arthrosphaerae* |  | 1 |  |  |
| *Chryseobacterium indologenes* |  | 2 |  |  |
| *Chryseobacterium* sp. |  | 2 |  | 1 |
| *Elizabethkingia* sp. |  | 1 |  |  |
| *Enterobacter hormaechei* |  | 2 |  |  |
| *Enterobacter mori* |  | 1 |  |  |
| *Enterobacter* sp. |  | 2 |  |  |
| *Escherichia coli* |  | 4 |  |  |
| *Neisseria subflava* |  | 5 |  |  |
| *Ochrobactrum anthropi* |  | 1 |  |  |
| *Pseudomonas aeruginosa* |  | 1 |  |  |
| *Pseudomonas atacamensis* |  | 1 |  |  |
| *Pseudomonas fulva* |  | 1 |  |  |
| *Pseudomonas nitroreducens* |  | 2 |  |  |
| *Pseudomonas oryzihabitans* |  |  |  | 1 |
| *Pseudomonas rhodesiae* |  | 2 |  |  |
| *Pseudomonas* sp. |  | 12 |  | 2 |
| *Roseomonas cervicalis* |  | 1 |  |  |
| *Sphingobacterium multivorum* |  | 1 |  |  |
| *Sphingobacterium spiritivorum* |  | 1 |  |  |
| *Stenotrophomonas maltophilia* |  | 24 |  | 4 |
| *Stenotrophomonas pavanii* |  | 4 |  |  |
| *Stenotrophomonas* sp. |  | 4 |  | 1 |
| Total |  | 131 |  | 13 |
