# Supplementary material for: The oral cavity is a potential reservoir of gram-negative antimicrobial-resistant bacteria, which are correlated with ageing and the number of teeth
Source: Heliyon. 2024 Oct 28;10(21):e39827. doi: 10.1016/j.heliyon.2024.e39827 (PMC11565020; doi:10.1016/j.heliyon.2024.e39827)
Supplement: Multimedia component 3 [file mmc3.docx]

**Table S3. Number of patients with detection of *Acinetobacter*, *Pseudomonas*, and *Stenotrophomonas* in the tongue microbiota in the 16S rRNA gene sequencing analysis.**

|  | **Number of patients (%)** | | |
| --- | --- | --- | --- |
|  | **GN-ARB**  **not isolated**  **(n = 389)** | **GN-ARB**  **isolated**  **(n = 85)** | ***P*-value^a^** |
| *Acinetobacter* | 2 (0.5) | 4 (4.7) | 0.011 |
| *Pseudomonas* | 4 (1.0) | 3 (3.5) | 0.11 |
| *Stenotrophomonas* | 0 (0.0) | 7 (8.2) | <0.001 |

^a^Fisher’s exact test.
